# A causal network analysis in an observational study identifies metabolomics pathways influencing plasma triglyceride levels

**DOI:** 10.1007/s11306-016-1045-2

**Published:** 2016-05-11

**Authors:** Azam Yazdani, Akram Yazdani, Ahmad Saniei, Eric Boerwinkle

**Affiliations:** 1 Human Genetics Center, University of Texas Health Science Center, 1200 Pressler Street, Houston, TX 77030 USA; 2 Department of Software Systematics, 14482 Potsdam, Germany

**Keywords:** Metabolomics, Causal network, Genome DAG, Triglyceride levels, Confounder, Direct effect

## Abstract

**Introduction:**

Plasma triglyceride levels are a risk factor for coronary heart disease. Triglyceride metabolism is well characterized, but challenges remain to identify novel paths to lower levels. A metabolomics analysis may help identify such novel pathways and, therefore, provide hints about new drug targets.

**Objectives:**

In an observational study, causal relationships in the metabolomics level of granularity are taken into account to distinguish metabolites and pathways having a direct effect on plasma triglyceride levels from those which are only associated with or have indirect effect on triglyceride.

**Method:**

The analysis began by leveraging near-complete information from the genome level of granularity using the GDAG algorithm to identify a robust causal network over 122 metabolites in an upper level of granularity. Knowing the metabolomics causal relationships, we enter the triglyceride variable in the model to identify metabolites with direct effect on plasma triglyceride levels. We carried out the same analysis on triglycerides measured over five different visits spanning 24 years.

**Result:**

Nine metabolites out of 122 metabolites under consideration influenced directly plasma triglyceride levels. Given these nine metabolites, the rest of metabolites in the study do not have a significant effect on triglyceride levels at significance level alpha = 0.001. Therefore, for the further analysis and interpretations about triglyceride levels, the focus should be on these nine metabolites out of 122 metabolites in the study. The metabolites with the strongest effects at the baseline visit were arachidonate and carnitine, followed by 9-hydroxy-octadecadenoic acid and palmitoylglycerophosphoinositol. The influence of arachidonate on triglyceride levels remained significant even at the fourth visit, which was 10 years after the baseline visit.

**Conclusion:**

These results demonstrate the utility of integrating multi-omics data in a granularity framework to identify novel candidate pathways to lower risk factor levels.

**Electronic supplementary material:**

The online version of this article (doi:10.1007/s11306-016-1045-2) contains supplementary material, which is available to authorized users.

## Introduction

Plasma triglyceride levels are a risk factor for coronary heart disease (Hokanson and Austin 1996; Fontbonne et al. 1989). Triglyceride levels are negatively correlated with high density lipoprotein (HDL)-cholesterol levels, and considerable attention has been placed on therapeutics that raise HDL-cholesterol levels (Nicholls et al. 2011). Recent Mendelian randomization studies, however, have cast doubt on the role of HDL-cholesterol as a causative risk factor for CHD (Voight et al. 2012) and refocused effort on triglyceride levels. Triglyceride metabolism is well characterized, but challenges remain to identify novel paths to lower plasma triglyceride levels. A metabolomics analysis may help identify such novel pathways and, therefore, provide hints about new drug targets. Metabolomics provides a powerful tool to better understand cellular and organismal lipid metabolism (German et al. 2007).

The metabolome consists of hundreds of correlated compounds, and there is considerable information in the metabolomics network that is not contained in the levels of individual metabolites (Gao et al. 2010; Karnovsky et al. 2012). Analyses of individual metabolites do not provide information about likely targets of intervention because of co-linearity among the metabolites, confounders among metabolites, and likely untoward effects. Even by fitting triglyceride on all metabolites simultaneously, the coefficients cannot be interpreted as true effects due to collinearity. One approach to directly incorporate relationships among the metabolome during the analysis of risk factor levels (e.g. triglycerides) is to consider networks. The importance of considering biologic networks in relation to human disease have been widely discussed (e.g. Vidal et al. 2011; Barabasi and Oltvai 2004). In observational studies, there have been few practical applications. Some examples of using genomic variations to identify causal relationships among phenotypes in observational studies are found in Zhu et al. (2007), Gomez-Cabrero et al. (2014), Barupal et al. (2012), Inouye et al. (2010) and Schadt et al. (2005).

In this study, we integrate genomics, metabolomics, and triglyceride risk factor of disease using the GDAG algorithm. By integrating data from different granularities and using knowledge about causal relationship between granularities, we are able to achieve causal inference that is less susceptible to confounding by hidden variables and, as a result, estimate robust causal networks which are well anchored to domain knowledge. The GDAG algorithm extracts information across the genome to create strong instrumental variables for generating robust causal relationships among metabolites (Yazdani et al. 2016b). Extracting information across the genome provides sufficient and reliable information so that there is no need to limit the number of metabolites under consideration. Using the genome granularity to identify causal relationships among metabolomics level of granularity is based on Mendelian randomization, an established approach to identify causal relationships. The algorithm then directly incorporates metabolomics causal relationships during the analysis of triglyceride levels. Taking the metabolomics causal network into account and then finding metabolites with direct effects on triglyceride levels, we can identify confounders at metabolomics level and overcome collinearity among metabolites. Therefore, we can identify novel intervention targets likely to lower plasma triglyceride levels and perhaps disease risk. Details of having causal inference in observational studies are provided in online Appendix 1 (Dawid 2007; Rubin 2005; Pearl 2009; Yazdani and Boerwinkle 2014). For terminology in causality see Yazdani and Boerwinkle (2015).

## Methods

### Study sample and triglyceride measurements

Genomic, metabolomics and triglyceride data were available on a subset of the Atherosclerosis Risk in Communities (ARIC) study, a biracial longitudinal cohort of 15,792 middle-aged individuals who were randomly sampled from four US communities and have been measured for multiple risk factor phenotypes related to health and chronic disease. A detailed description of the ARIC study design and methods has been published elsewhere (The ARIC Investigators 1989). The data presented here includes 2479 African-American individuals from the Jackson, MS field center having genomics, metabolomics, and fasting plasma triglyceride levels.

Common single nucleotide polymorphisms (SNPs) were genotyped using the Affymetrix platform (version 6.0) consisting of 1,034,945 common variants spread across the genome. We reduce the number of SNPs by considering the fact that some SNPs are nearly perfectly correlated (>0.80) with others, so that one SNP can thereby serve as a proxy for many others in the analysis. To determine a proxy, we use hierarchical clustering and the squared correlation measure of linkage disequilibrium, for more details see Yazdani and Dunson (2015). Metabolomics levels were measured on fasting serum using a combination of gas chromatography, liquid chromatography, and mass spectroscopy. The analysis presented here consists of 122 reliably measured metabolites with low levels of missing data and normally distributed after transformation.

Triglyceride levels were measured from blood plasma collected in the fasting state. Triglyceride levels were measured enzymatically at five different visits during 24 years of follow-up; the serum metabolome was measured at visit 1. The number of individuals with metabolomics data and examined at each visit is, respectively, 2479, 1920, 1629, 1398 and 700. After winsorising and taking the logarithm, serum triglyceride levels followed a normal distribution, which was the variable analyzed here.

### Metabolomics and triglyceride causal network

In this study, we applied the GDAG algorithm, which is a constraint-based algorithm, to generate a robust causal network over 122 metabolites under consideration using genome information. The GDAG algorithm first identifies a topology (a network without direction) over the variables. A missing edge between two nodes means the two corresponding variables are independent given the rest of variables in the model. An edge between two nodes means that the two corresponding variables are dependent given the rest of the variables under consideration. The only tuning parameter in the GDAG algorithm is significance level, alpha, which based on preliminary analyses is set to 0.001. Directionality over the topology which have causal interpretation is then identified using strong instrumental variables created by extracting information across the genome. The genome strong instrumental variables identify causal relationships over the 122 metabolites under consideration. Online Appendix 2 details this multistep procedure and online Appendix 3 visualizes the metabolomics causal relationships.

Next, we integrate metabolomics granularity and triglyceride variable using the GDAG algorithm. Given the metabolomics causal network from the previous step, we enter triglyceride levels into the model. We assess the direct effect of each metabolite on triglyceride levels given the metabolomics causal network which directly takes into account confounders and allows for causal interpretations, see online Appendix 1 (Yazdani et al. 2016a).

## Results

**Fig. 1:**
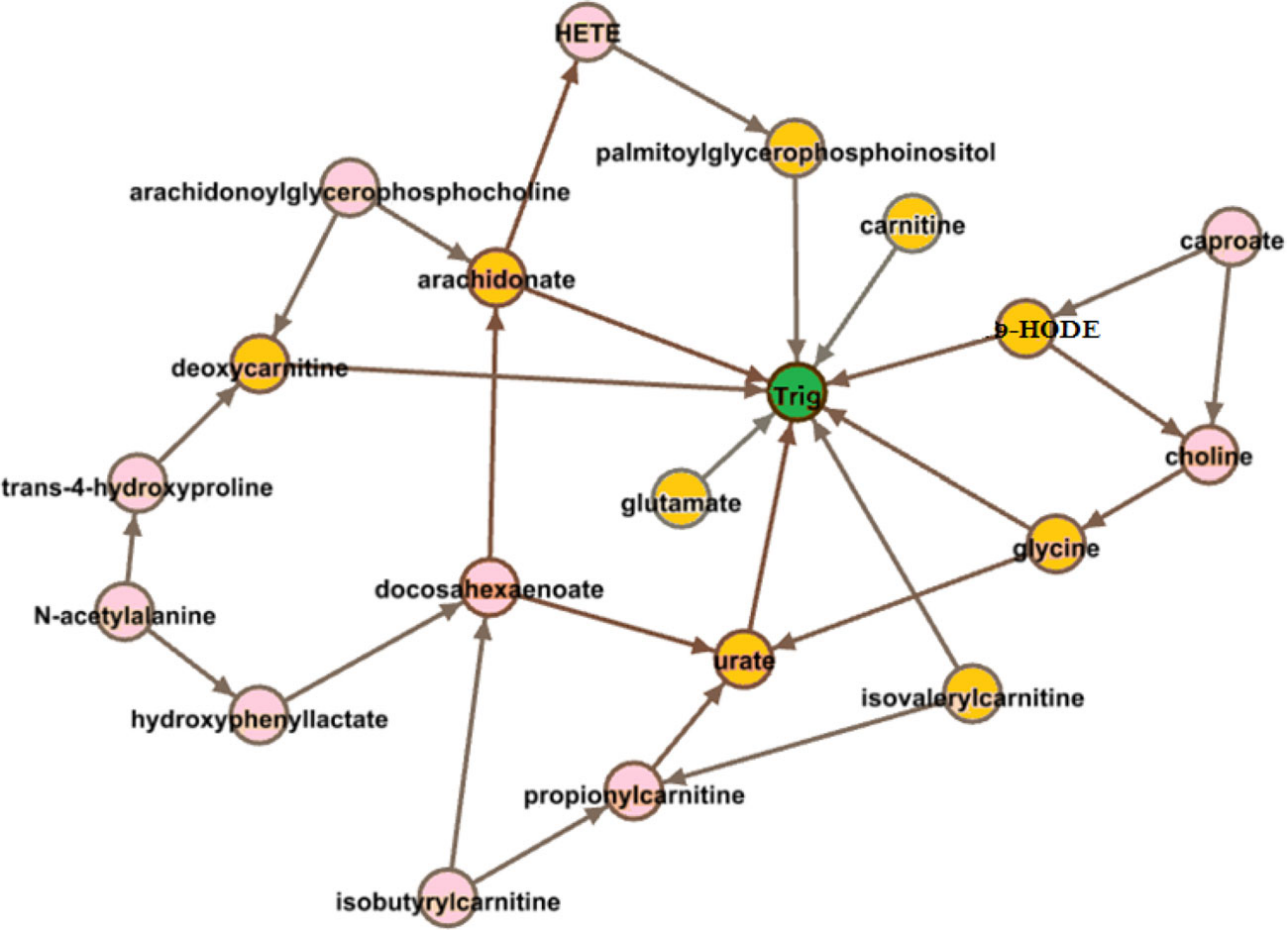
The metabolomics-triglyceride statistical causal network. This network represents metabolites with direct effect (shown in *orange*) and indirect effect (shown in *pink*) on triglyceride (shown in *green*) (Color figure online)

Among the 122 metabolites in this analysis, nine metabolites have direct effects on triglyceride levels at visit 1. Figure 1 depicts the relationship among the nine metabolites with direct effects and ten metabolites with indirect effect on triglyceride levels. These nine metabolites directly influenced triglyceride levels after conditioning on the other metabolites in the study and are circled in red in online Appendix 3 to show their location in the overall metabolomics network. Given these nine metabolites, the rest of metabolites in the study do not have a significant effect on triglyceride levels at significance level alpha = 0.001.

We next investigated the effect of the nine metabolites measured at the baseline examination (i.e. visit 1) on fasting plasma triglyceride levels measured at each study visit. Table 1 provides the names and select biologic characteristics of the nine metabolites ordered by the *p* value testing the relationship of each with triglyceride levels at visit 1. Not surprisingly, the most common super-pathway is lipid metabolism. None of the nine metabolites are long chain fatty acids. The metabolite with the strongest relationship was arachidonate, a derivative of arachidonic acid. The ARIC study has had multiple examination from 3 to 15 years apart, and the metabolomics data were collected at the baseline examination (i.e. visit 1). The baseline metabolites with the strongest relationship with triglyceride were arachidonate and carnitine, followed by 9-hydroxy-octadecadenoic acid (9-HODE) and palmitoylglycerophosphoinositol. It is of note that the baseline aracidonate metabolite had a profound relationship with triglyceride levels at each of the first four visits. There was no metabolite that significantly influenced triglyceride levels after 24 years at visit 5 (data not shown).

**Table 1:** Metabolites with direct effects on triglyceride levels ordered by the *p* value at visit 1

Metabolite name	Super-pathway	Sub-pathway	Total effect *p* value			
			V1	V2	V3	V4
Arachidonate	Lipid	Long chain fatty acid	2.3e−17	1e−10	7.3e−9	3.2e−9
Carnitine	Lipid	Carnitine metabolism	1.4e−11	8.7e−3	6.5e−3	3.6e−3
9-HODE	Lipid	Fatty acid, monohydroxy	1.4e−7	2.3e−4	5.6e−3	2.7e−3
Palmitoylglycerophos-phoinositol	Lipid	Lysolipid	1.6e−6	1.4e−3	7.6e−3	5.9e−3
Urate	Nucleotide	Purine metabolism, urate metabolism	2.2e−5	8.3e−5	4.7e−4	5.9e−3
Isovalerylcarnitine	Amino acid	Valine, leucine and isoleucine metabolism	2.0e−4	7.6e−3	9.5e−2	8.7e−2
Glycine^*^(–)	Amino acid	Glycine, serine and threonine metabolism	3.4e−3	4.7e−3	2.4e−3	8.6e−3
Deoxycarnitine^*^(–)	Lipid	Carnitine metabolism	1.1e−3	1.2e−3	4.7e−3	5.3e−3
Glutamate^*^	Amino acid	Glutamate metabolism	4.1e−3	2.3e−3	2.4e−3	2.1e−3

– Metabolite with an inverse relationship with triglyceride levels

* Metabolite with no significant effect at level 0.001 after adjusting for BMI

We measured the effect of each metabolite on baseline triglyceride levels given the overall metabolomics network. Details about effect measurement are provided in online Appendix 1. The results are shown in Table 2. To facilitate comparison across time and among metabolites, these total effects are presented in standard deviation units. Glycine, deoxycarnitine and glutamate had nominal effects on triglycerides, and these effects were not significant after adjusting for BMI.

**Table 2:** Metabolites with direct effect on triglycerides ordered by their effect sizes at visit 1

Metabolite name	Super-pathway	Sub-pathway	Total effect^a^ (SE)			
			V1	V2	V3	V4
Arachidonate	Lipid	Long chain fatty acid	0.17 (0.03)	0.13 (0.02)	0.16 (0.05)	0.18 (0.03)
Carnitine	Lipid	Carnitine metabolism	0.15 (0.04)	0.09 (0.03)	0.08 (0.03)	0.06 (0.04)
9-HODE	Lipid	Fatty acid, monohydroxy	0.12 (0.03)	0.12 (0.04)	0.07 (0.02)	0.10 (0.06)
Palmitoylglycerophos-phoinositol	Lipid	Lysolipid	0.10 (0.01)	0.07 (0.03)	0.01 (0.00)	0.06 (0.01)
Urate	Nucleotide	Purine metabolism, urate metabolism	0.09 (0.01)	0.09 (0.01)	0.10 (0.03)	0.07 (0.01)
Isovalerylcarnitine	Amino acid	Valine, leucine and isoleucine metabolism	0.09 (0.02)	0.08 (0.01)	0.03 (0.03)	0.05 (0.02)
Glycine	Amino acid	Glycine, serine and threonine metabolism	−0.09 (0.02)	−0.07 (0.01)	−0.11 (0.04)	−0.07 (0.03)
Deoxycarnitine	Lipid	Carnitine metabolism	−0.08 (0.03)	0.03 (0.02)	0.11 (0.02)	0.09 (0.01)
Glutamate	Amino acid	Glutamate metabolism	0.07 (0.02)	0.05 (0.01)	0.04 (0.00)	0.03 (0.02)

^a^Effect sizes measured in standard deviation units to facilitate comparison

## Discussion

We analyzed the relationship between a causal network among 122 serum metabolites and plasma triglyceride levels with the long-term purpose of identify potential points of intervention within the metabolome, which may translate into downstream lowering of triglyceride levels and possibly reduced risk of cardiovascular disease. In this analysis, causal inference in an observational study was facilitated by incorporation of genomic information, which provided robust direction relationships among the metabolites. Based on these analyses, we identified nine metabolites with a significant direct effect on triglyceride levels. Given these nine metabolites, the rest of metabolites in the study do not have a significant effect on triglyceride levels at significance level alpha = 0.001. Therefore, in this manuscript, we have focused on the presentation and interpretation of these nine metabolites. Five of the nine were in the lipid metabolism super pathway, and three of the nine were in the amino acid super pathway. The four metabolites with the largest effect on triglyceride were in the lipid metabolism super pathway. The two metabolites with the largest effects include arachidonate and carnitine. The effects of arachidonate on triglycerides remained significant 10 years after the original measurement of the metabolome. Having a causal network between the metabolome and triglycerides, as opposed to only correlations, allows one to identify potential points of intervention, either pharmacologic or genetic, that would be predicted to alter triglyceride levels. Traditionally, such causal inference would only be possible in a clinical trials setting, but the GDAG approach implemented here permits such predictions in an observational setting.

In the current analysis, by integrating data from different biological hierarchies, we were able to derive causal inference that is less susceptible to confounding by hidden variables and, as a result, estimate robust causal network over metabolites in the analysis. We then applied the metabolomics network to find metabolites with direct effect on triglyceride levels. Multiple previous studies have used both a priori defined and data-driven networks to analyze metabolomics data (Gao et al. 2010; Karnovsky et al. 2012; Grapov et al. 2015; Bartel et al. 2013; Krumsiek et al. 2011). To our knowledge, this is the first application that connects genomics, metabolomics, and risk factors in an observational setting.

The metabolite with the largest effect on triglycerides is arachidonate, which in vivo is often esterified to a glycerophospholipid. Arachidonate is released from the phospholipid by phospholipase A2-catalzyed hydrolysis. Lp-PLA2 along with triglycerides are major determinants of small dense LDL, a major cardiovascular disease risk factor. In hamster feeding experiments, addition of arachidonic acid to the diet increases blood triglyceride levels (Whelan et al. 1995). In humans, there is considerable interest in the role of brain arachidonic acid in membrane function and as a precursor for eicosanoids. Infants fed arachidonic acid as a triglyceride or phospholipid had increase brain and other organ arachidonic acid accretion (Wijendran et al. 2002). The causal network among metabolites allows a more nuanced analysis of their effect on triglyceride levels compared to a simple pairwise analysis. Looking at Fig. 1, arachidonate also influences triglycierides indirectly via its relationship with palmitoylglycerophosphoinositol. These multiple paths magnify the effect any intervention on archidonate would have on triglyceride levels.

The metabolite with the second largest predicted impact on triglyceride levels was carnitine. Carnitine aids in the transport of fatty acids into the mitochondrial matrix during the break down of lipid on its way to making energy. There is already considerable literature that dietary supplementation with carnitine altering blood triglyceride levels in a number of settings (Bell and DeLucia 1984; Vacha et al. 1983; Künnert et al. 1983). In some ways then, carnitine serves as an internal positive control for the current study, and it is reassuring that the GDAG approach identified a direct relationship between carnitine and triglycerides.

9-HODE is a product of free radical oxidation of linoleic acid and is an agonist of PPARγ. 9-HODE is a biomarker of oxidative stress (Fruhwirth et al. 2007). There are many sources of 9-HODE, including lipoprotein lipase-mediated lipolysis of triglycerides (Wang et al. 2009). In addition, there are multiple paths by which 9-HODES may be involved in the development of atherosclerosis and risk of CHD, including oxidized LDL, HDL metabolism and PPARγ action. These same paths and players are likely involved in the relationship between 9-HODES and plasma triglyceride levels. There are interventions that would directly impact 9-HODE. PPARγ agonists decrease HODE levels in obese diabetic patients (Popkin and Gordon-Larsen 2004). To our knowledge, the effect of up or down regulation of lipoprotein lipase or naturally occurring lipoprotein lipase deficiency on 9-HODE levels have not been reported.

There is little available about the function of palmitoylglycerophosphoinositol, and nothing to our knowledge about its relationship with triglycerides. The data presented here, therefore, shed needed light on this portion of the human serum metabolome. In contrast, there is a considerable knowledgebase on the relationship between uric acid and triglycerides. Large observational studies support a positive relationship between uric acid levels and triglyceride levels, and some have argued this is due to a shared effect of dietary sugar intake (Hofmann and Tschöp 2009). In addition, smaller intervention studies support a positive relationship. For example, use of fenofibrate, a PPARα agonist, reduces both blood triglyceride and uric acid levels, presumably through increased fatty acid β oxidation (Patterson et al. 2009). The data presented here from causal inference of the human serum metabolome further supports this important link.

### Concluding remarks

In summary, the analyses and results presented here are significant for three reasons. First, they document the utility of the GDAG algorithm for generating robust and informative networks among the components of the serum metabolome and reduce the number of metabolites that we need to focus on for further analysis of the risk factor under consideration. Second, they used the metabolomics network as a starting point to analyze plasma triglyceride levels across multiple visits in a large sample of individuals, and identified nine metabolites with a direct effect on triglyceride levels. And third, these results move us one step closer to identifying novel points or pathways of intervention to lower plasma triglyceride levels and possible cardiovascular disease risk.

## Electronic supplementary material

Below is the link to the electronic supplementary material.

Supplementary material 1 (DOCX 712 kb)

## References

[CR1] Barabasi AL, Oltvai ZN (2004). Network biology: Understanding the cell’s functional organization. Nature Reviews Genetics.

[CR2] Bartel J, Krumsiek J, Theis FJ (2013). Statistical methods for the analysis of high-throughput metabolomics data. Computational and Structural Biotechnology Journal.

[CR3] Barupal DK (2012). MetaMapp: Mapping and visualizing metabolomic data by integrating information from biochemical pathways and chemical and mass spectral similarity. BMC Bioinformatics.

[CR4] Bell FP, DeLucia A (1984). An inverse relationship between plasma carnitine and triglycerides in selected Macaca arctoides and Macaca nemistrina fed a low-fat chow diet. Comparative Biochemistry and Physiology Part B: Comparative Biochemistry.

[CR5] Dawid A.P., (2007) *Fundamentals of statistical causality*. Research report 279, Department of statistical science, University College London.

[CR6] Fontbonne A (1989). Hypertriglyceridaemia as a risk factor of coronary heart disease mortality in subjects with impaired glucose tolerance or diabetes. Diabetologia.

[CR7] Fruhwirth GO, Loidl A, Hermetter A (2007). Oxidized phospholipids: From molecular properties to disease. Biochimica et Biophysica Acta (BBA)-Molecular Basis of Disease.

[CR8] Gao J (2010). Metscape: A Cytoscape plug-in for visualizing and interpreting metabolomic data in the context of human metabolic networks. Bioinformatics.

[CR9] German JB (2007). Lipidomics and lipid profiling in metabolomics. Current Opinion in Lipidology.

[CR10] Gomez-Cabrero D (2014). Data integration in the era of omics: Current and future challenges. BMC Systems Biology.

[CR11] Grapov D, Wanichthanarak K, Fiehn O (2015). MetaMapR: Pathway independent metabolomic network analysis incorporating unknowns. Bioinformatics.

[CR12] Hofmann SM, Tschöp MH (2009). Dietary sugars: A fat difference. The Journal of Clinical Investigation.

[CR13] Hokanson JE, Austin MA (1996). Plasma triglyceride level is a risk factor for cardiovascular disease independent of high-density lipoprotein cholesterol level: A metaanalysis of population-based prospective studies. Journal of Cardiovascular Risk.

[CR14] Inouye M, Kettunen J, Soininen P, Silander K, Ripatti S, Kumpula LS, Peltone L (2010). Metabonomic, transcriptomic, and genomic variation of a population cohort. Molecular Systems Biology.

[CR16] Karnovsky A (2012). Metscape 2 bioinformatics tool for the analysis and visualization of metabolomics and gene expression data. Bioinformatics.

[CR17] Krumsiek J, Suhre K, Illig T, Adamski J, Theis FJ (2011). Gaussian graphical modeling reconstructs pathway reactions from high-throughput metabolomics data. BMC Systems Biology.

[CR18] Künnert B (1983). Metabolic triglyceride storage disorders. A report of 2 cases of systemic carnitine deficiency. Zentralblatt fur allgemeine Pathologie und pathologische Anatomie.

[CR19] Nicholls SJ (2011). Effects of the CETP inhibitor evacetrapib administered as monotherapy or in combination with statins on HDL and LDL cholesterol: A randomized controlled trial. JAMA.

[CR20] Patterson AD (2009). Human urinary metabolomic profile of PPARα induced fatty acid β-oxidation. Journal of Proteome Research.

[CR21] Pearl J (2009). Causality: Models, reasoning, and inference.

[CR22] Popkin BM, Gordon-Larsen P (2004). The nutrition transition: Worldwide obesity dynamics and their determinants. International Journal of Obesity.

[CR23] Rubin DB (2005). Causal inference using potential outcomes: Design, modeling, decisions. Journal of the American Statistical Association.

[CR24] Schadt Eric E (2005). An integrative genomics approach to infer causal associations between gene expression and disease. Nature Genetics.

[CR25] The ARIC Investigators (1989). The Atherosclerosis Risk in Communities (ARIC) Study: Design and objectives. The ARIC investigators. American Journal of Epidemiology.

[CR26] Vacha Gian Maria (1983). Favorable effects of L-carnitine treatment on hypertriglyceridemia in hemodialysis patients: Decisive role of low levels of high-density lipoprotein-cholesterol. The American Journal of Clinical Nutrition.

[CR27] Vidal M, Cusick ME, Barabasi AL (2011). Interactome networks and human disease. Cell.

[CR28] Voight BF (2012). Plasma HDL cholesterol and risk of myocardial infarction: A mendelian randomisation study. The Lancet.

[CR29] Wang L (2009). Triglyceride-rich lipoprotein lipolysis releases neutral and oxidized FFAs that induce endothelial cell inflammation. Journal of Lipid Research.

[CR30] Whelan J (1995). Evidence that dietary arachidonic acid increases circulating triglycerides. Lipids.

[CR31] Wijendran V (2002). Efficacy of dietary arachidonic acid provided as triglyceride or phospholipid as substrates for brain arachidonic acid accretion in baboon neonates. Pediatric Research.

[CR32] Yazdani A, Dunson DB (2015). A hybrid Bayesian approach for genome-wide association studies on related individuals. Bioinformatics.

[CR33] Yazdani A, Boerwinkle E (2014). Causal inference at the population level. International Journal of Research in Medical Sciences.

[CR34] Yazdani A, Boerwinkle E (2015). Causal inference in the age of decision medicine. Journal Data Mining Genomics Proteomics.

[CR35] Yazdani A, Yazdani A, Boerwinkle E (2016). Conceptual aspects of causal networks in an applied context. Journal Data Mining Genomics Proteomics.

[CR36] Yazdani A, Yazdani A, Samiei A, Boerwinkle E (2016). Generating a robust statistical causal structure over 13 cardiovascular disease risk factors by data integration. Journal of Biomedical Informatics.

[CR37] Zhu J, Wiener M, Zhang C, Fridman A, Minch E, Lum P, Sachs J, Schadt E (2007). Increasing the power to detect causal associations by combining genotypic and expression data in segregating populations. PLoS Computational Biology.

