# Supplementary material for: A causal network analysis in an observational study identifies metabolomics pathways influencing plasma triglyceride levels
Source: Metabolomics. 2016 May 11;12(6):104. doi: 10.1007/s11306-016-1045-2 (PMC4869741; doi:10.1007/s11306-016-1045-2)
Supplement: Supplementary file 1 — Supplementary material 1 (DOCX 712 kb) [file 11306_2016_1045_MOESM1_ESM.docx]

**Appendix 1**

**Causal Inference in Observational Study**

In observational studies, where intervention is not possible, causal inference is derived by identifying the assignment mechanism (AM), i.e. causal relationships, underlying the observations (Rubin, 2005; Pearl, 2009; Dawid, 2007; Yazdani, 2014). The *AM* represents how different levels of a response variable are assigned and which covariates are involved. The illumination of the *AM* is carried out using knowledge about response variables ($K_{R}$) and is required for any causal inference. This assumption is formalized by $AM(K_{R})$and is called causal parameter (Yazdani and Boerwinkle, 2014). Any causal quantity is implicitly or explicitly conditioned on the causal parameter$AM(K_{R})$. The causal parameter represents the necessity of illumination of the *AM* by$K_{R}$.

In this study, the *AM* is illuminated by the fact that genomic variation in a deeper granularity is a causal factor for variations in the metabolomics level of granularity and not the other way around. This is the applied knowledge about metabolite response variables ($K_{R}$) to illuminate the *AM*. Here, the *AM* or causal relationships behind observations are formalized by a directed acyclic graph. We then graphically identify confounders among covariates to measure causal effect sizes. For the definition of confounders and covariates see (Yazdani and Boerwinkle, 2015).

**Causal Effect Measurement**

We measured the effect of each metabolite on baseline triglyceride levels given the overall metabolomics network by

$RF_{Trig}|AM(K_{R})=\sum_{i\in pa(Trig)} \beta_{i}M_{i}+U$,

where $M_{i}$stands for a metabolite corresponding node *i* and$pa(Trig)$stands for nodes with an arrow in node *Trig* in the generated causal network. The node *Trig* corresponds the risk factor variable triglyceride.$AM(K_{R})$stands for the causal parameter and conditioning on the causal parameter means the assignment mechanism (AM)/causal relationships behind observations are illuminated by gathering knowledge about response variables. By illumination of the *AM*, we can identify confounders at metabolomics level and assume$U\perp M_{i}$. As a result, we can estimate the effect of a specific metabolite on triglyceride levels. Therefore, the coefficients$\beta_{i}$have causal interpretations. For details about causal effect measurement see (Yazdani et al., 2016b).

**Appendix 2**

**The GDAG Algorithm** (a constraint-based algorithm)

The GDAG algorithm: The GDAG algorithm (Yazdani et al. 2016a) uses genome-wide principal components as strong instrumental variables to identify a stable network over the metabolites. To extract information across the genome, **first**, we start by reducing the number of SNPs by considering the fact that some SNPs are nearly perfectly correlated (>0.80) with others, so that one SNP can thereby serve as a proxy for many others in the analysis. To determine a proxy, we use hierarchical clustering and a measure of linkage disequilibrium (Yazdani and Dunson, 2015). **Second**, the genomic information was summarized using the set of principal components applied to each chromosome. Since chromosomes are independent, we can apply principal component analysis to each chromosome. **Third**, the set of principal components responsible for more than 90% of the variation over each chromosome was selected. Over all of the chromosomes, 788 principal components were selected to identify a causal network over 122 metabolites. Based on preliminary analyses, the tuning parameter in the GDAG algorithm was set to 0.001. The GDAG algorithm first generated a network topology over the 910 variables: 788 selected genome-wide PCs and 122 metabolites. The network topology represents the Markov conditions over the variables. This step determines the genome-wide principal components that are significantly connected to each metabolite given other metabolites in the model. Since the direction of effect is from the genome level of granularity to the phenotype level of granularity, the GDAG algorithm overlays the direction from the PCs to the metabolites and finally determines directionality among the metabolites. Note that the primary aim is identification of a causal network over the set of metabolites and the genome information in the deeper granularity is a tool to aid in identifying directionality among the metabolites in the upper level. Therefore, after the metabolomics network is identified the genome PCs are deleted from the network in Appendix3.

Given the metabolomics causal network, we next enter the triglyceride variable into the model to find metabolites with direct influence on the triglyceride levels.

**Appendix 3**

Metabolomics causal Network

**
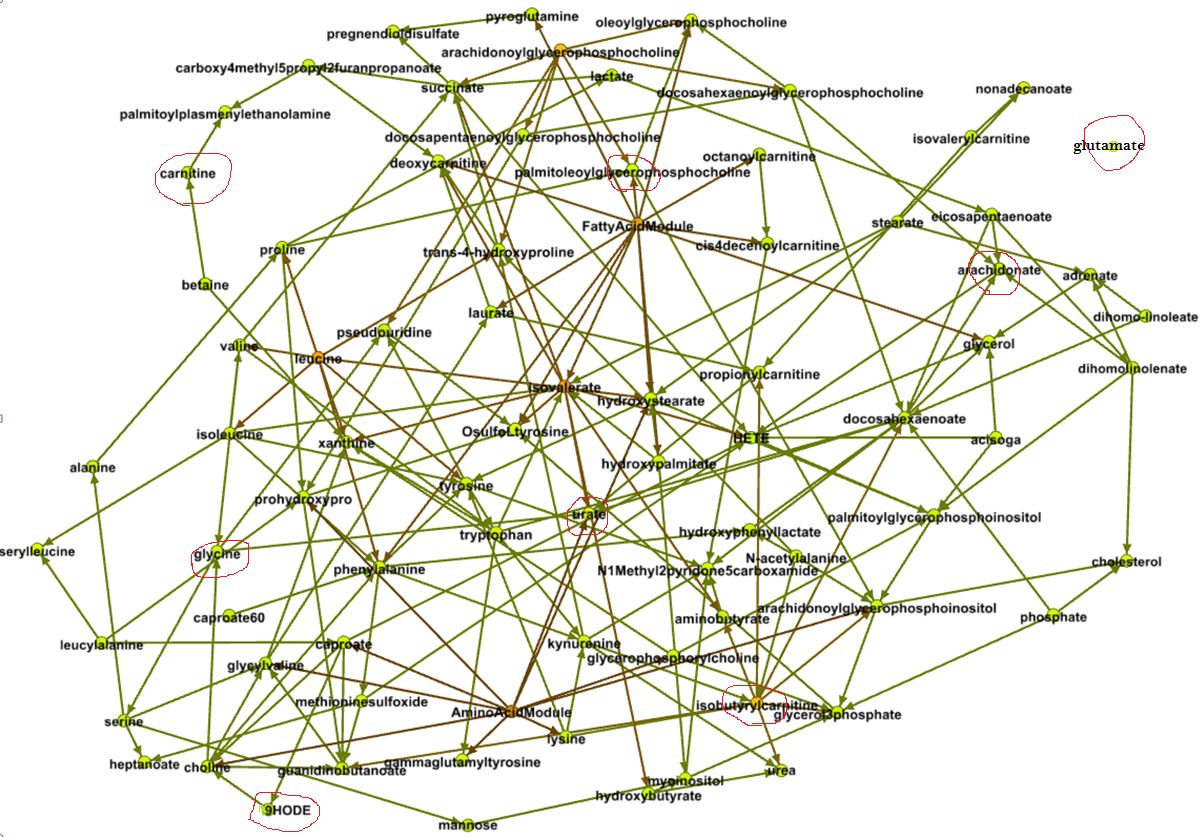
**

**Figure** 1: Metabolomics Causal Network over 122 serum metabolites among ARIC African-Americans. The metabolites with a red circle around directly influence on triglyceride levels. This network is generated using extracted information from genome. The genome predictors are not depicted since our interest is the metabolomics network.
